# Chrysin Pretreatment Enhances BMSC Therapeutic Efficacy in Resolving Diabetic Wound Healing

**DOI:** 10.3390/biomedicines14040781

**Published:** 2026-03-30

**Authors:** Sicheng Li, Shengzhi Zhou, Tian Yang, Mosheng Yu, Yong Wang, Zhanyong Zhu

**Affiliations:** Department of Plastic Surgery, Renmin Hospital of Wuhan University, Wuhan 430060, China; m13808644226@163.com (S.L.); 2019305231064@whu.edu.cn (S.Z.); yangtia@whu.edu.cn (T.Y.);

**Keywords:** diabetic foot ulcer, wound healing, bone marrow mesenchymal stem cells, Chrysin, conditioned medium, inflammation

## Abstract

**Background**: Diabetic wounds represent a major clinical challenge due to persistent inflammation, oxidative stress, and impaired angiogenesis. Bone marrow mesenchymal stem cells (BMSCs) have strong regenerative potential, and their therapeutic effects and optimization strategies for diabetic wounds warrant further exploration. **Objective**: This study aimed to improve the therapeutic efficacy of BMSCs in diabetic wound healing via chrysin pretreatment, as well as to evaluate the healing capacity and molecular mechanisms of the derived chrysin-pretreated BMSC-conditioned medium (Chrysin-CM). **Methods**: BMSCs were pretreated with 1 μM chrysin for 48 h to generate Chrysin-CM. The therapeutic effects were evaluated in vitro by analyzing the proliferation, migration, and matrix synthesis of human umbilical vein endothelial cells (HUVECs) and human skin fibroblasts (HSFs) under high-glucose (HG) conditions. In vivo, a diabetic mouse model with full-thickness excisional wounds was established and treated topically with Chrysin-CM. Transcriptomic sequencing and immune infiltration analysis of wound tissues were performed on day 14 in order to investigate the underlying mechanisms. **Results**: Chrysin pretreatment significantly enhanced the functional activity of BMSCs, accompanied by increased proliferative capacity and accelerated cell cycle progression. In vitro, Chrysin-CM demonstrated superior efficacy, robustly protecting HUVECs and HSFs from HG-induced dysfunction. In vivo, Chrysin-CM significantly accelerated wound closure, re-epithelialization, and neovascularization compared to the control. Mechanistically, RNA sequencing (RNA-seq) revealed that Chrysin-CM induced multi-level remodeling, characterized by reduced inflammatory gene expression and immune cell infiltration, along with the upregulation of regenerative genes and alternative splicing events. **Conclusions**: Chrysin successfully improved the therapeutic efficacy of the BMSC secretome in wound healing. Chrysin-CM effectively accelerated diabetic wound healing by actively resolving chronic inflammation and promoting angiogenesis and structural remodeling, thus providing a potential strategy for stem cell-based cell-free treatment for chronic diabetic wounds.

## 1. Introduction

Diabetic foot ulcer (DFU), one of the most severe and prevalent complications of diabetes mellitus, remains a major global public health challenge [1]. DFU remains the leading cause of non-traumatic lower limb amputation and is associated with high disability rates, substantial economic burden, and a markedly reduced quality of life. The difficulty in healing chronic diabetic wounds primarily stems from the complex pathological microenvironment, which is characterized by persistent low-grade inflammation, excessive oxidative stress, impaired angiogenesis, and severe disruption of extracellular matrix (ECM) synthesis and remodeling. Unfortunately, existing clinical treatments, such as debridement and wound dressings, often fail to satisfactorily address these fundamental pathological microenvironments [2]. Therefore, there is an urgent need to develop advanced regenerative strategies which are capable of restoring the wound microenvironment.

Among various regenerative therapies, bone marrow-derived mesenchymal stem cells (BMSCs) have emerged as promising candidates due to their self-renewal ability, multilineage differentiation potential, and the secretion of diverse bioactive factors [3]. The therapeutic effects of BMSCs are primarily mediated by their paracrine activity through the release of growth factors, cytokines, and extracellular vesicles that modulate inflammation and promote tissue regeneration. However, the direct clinical application of BMSCs remains challenging. In diabetic wounds characterized by ischemia, hyperglycemia, and chronic inflammation, transplanted BMSCs often exhibit low survival, functional decline, or apoptosis, which strongly limits their efficacy [4]. Furthermore, live-cell transplantation intrinsically carries safety and logistical hurdles, including potential tumorigenicity, immune rejection, uncontrolled ectopic differentiation, and high manufacturing costs.

To enhance the therapeutic effects of BMSC functions in the diabetic microenvironment, pharmacological priming strategies have been widely proposed as a feasible approach [5]. Some studies aimed to enhance BMSCs’ resistance to stress and optimize their secretory profiles in vitro before their clinical use. For instance, Otaibi et al. reported that melatonin-pretreated BMSCs significantly improved wound healing in rats compared with untreated BMSCs, accelerating tissue regeneration and reducing scarring by enhancing collagen synthesis, angiogenesis, and antioxidant capacity while suppressing inflammation [6]. Similarly, Cao et al. demonstrated that extracellular vesicles derived from hypoxia-preconditioned BMSCs promoted BMSC proliferation, human umbilical vein endothelial cell (HUVEC) angiogenesis, and peri-implant osteogenesis in diabetic rats through activation of the miR-106b-5p/HIF-1α pathway [7]. Consequently, the focus on functional enhancement has paved the way for cell-free therapies based on the BMSC secretome, offering a safe alternative by reducing the risks associated with stem cell transplantation.

Among potential priming agents, natural flavonoids have attracted increasing attention due to their broad bioactivity and low toxicity [8]. Chrysin, a dihydroxyflavone mainly found in honey, propolis, and passion flowers, exhibits potent antioxidant and anti-inflammatory properties. For instance, chrysin-incorporated composite scaffolds were non-cytotoxic to mouse MSCs and enhanced their proliferation and osteogenic differentiation, possibly through the downregulation of Runx2 co-repressors [9]. Furthermore, chrysin alleviated oxidative stress, improved BMSC viability, and promoted osteogenic differentiation via activation of the PI3K/AKT/Nrf2 pathway [10]. However, whether chrysin preconditioning can enhance the therapeutic function of BMSCs in the context of promoting diabetic wound healing remains unclear.

Based on these findings, this study aimed to investigate the potential of chrysin as a novel enhancer of the therapeutic efficacy of BMSCs in diabetic wound healing. Firstly, we evaluated the effects of chrysin pretreatment on the biological behaviors of BMSCs and assessed the protective and regenerative effects of their derived conditioned medium (CM) to assess the protective and regenerative effects on endothelial cells and fibroblasts under high-glucose (HG) conditions. Additionally, we established a streptozotocin (STZ)-induced diabetic mouse model to examine the in vivo therapeutic efficacy of chrysin-pretreated BMSC-conditioned medium (Chrysin-CM) and performed transcriptomic analysis to elucidate the underlying molecular mechanisms. This approach provides experimental evidence and a promising strategy for developing cell-free stem cell-based therapies to improve diabetic wound healing.

## 2. Materials and Methods

### 2.1. Isolation, Culture, and Identification of Mouse BMSCs

Primary BMSCs were isolated from the femurs of 3-week-old male C57BL/6 mice. The mice were purchased from Hubei Biont Biotechnology Co., Ltd. (Wuhan, China). All animal experiments were approved by the Institutional Animal Care and Use Committee of Renmin Hospital of Wuhan University (Approval No. WDRM-20250102C, approved on 11 Jan 2025). Briefly, mice were euthanized and their bone marrow cavities were flushed with serum-free Dulbecco’s Modified Eagle Medium/Nutrient Mixture F-12 (DMEM/F12, Cat. No. 11320; Gibco, Grand Island, NY, USA) to obtain cell suspensions. The suspension was filtered through a 70 μm cell strainer and centrifuged at 300 *g* × 5 min. Cells were then resuspended and cultured at 37 °C in a humidified atmosphere with 5% CO_2_ in DMEM/F12 supplemented with 20% fetal bovine serum (FBS; Cat. No. 10082147, Gibco, USA), 100 U/mL penicillin, and 100 μg/mL streptomycin. Non-adherent cells were removed after 48 h, and the medium was refreshed every 3 days. Cells at passage 3–6 were utilized for all subsequent experiments.

For immunophenotypic identification, BMSCs were trypsinized, washed, and incubated with fluorochrome-conjugated monoclonal antibodies against mouse antigens for 30 min at 4 °C in the dark. CD29, CD44, CD105, CD34, and CD45 antibodies were used (BD Biosciences, San Jose, CA, USA). Data were acquired using a flow cytometer and analyzed to confirm the expression of mesenchymal markers and the absence of hematopoietic markers.

For adipogenesis, cells were cultured in Adipogenic Differentiation Medium (Cat. No. MUBMD-90031, Cyagen Biosciences, Suzhou, China) for 14 days and stained with Oil Red O to visualize lipid droplets. For osteogenesis, cells were incubated in Osteogenic Differentiation Medium (Cat. No. MUBMD-90021, Cyagen Biosciences, China) for 21 days, followed by Alizarin Red S staining to detect calcium deposition. For chondrogenesis, cells were centrifuged to form high-density pellets and cultured in Chondrogenic Differentiation Medium (Cat. No. MUBMD-90041, Cyagen Biosciences, China) for 21 days, after which Alcian Blue staining was performed to verify the synthesis of sulfated proteoglycans.

### 2.2. Cell Culture

HUVECs (Cat. No. HUVEC-20001, Cyagen Biosciences, China) and human skin fibroblasts (HSFs, Cat. No. HXXFB-00001, Cyagen Biosciences, China) were obtained from Cyagen Biosciences. Cells were cultured in Dulbecco’s Modified Eagle Medium (DMEM) supplemented with 10% fetal bovine serum (FBS) at 37 °C in a humidified incubator with 5% CO_2_.

For the normal glucose condition, cells were maintained in low-glucose DMEM (Cat. No. 11885084, Gibco, USA) containing 5.6 mM (1 g/L) D-glucose. To establish the HG injury model, 30 mM (5.4 g/L) D-glucose (Cat. No. HY-B0389, MedChemExpress, Monmouth Junction, NJ, USA) was prepared by adding supplementary glucose into glucose-free DMEM (Cat. No. 11966025, Gibco, USA). The normal glucose and HG media were identical except for the glucose concentration. Cells were exposed to the HG condition for 72 h to induce glucose-related cellular injury before subsequent experiments.

### 2.3. Preparation of Conditioned Medium

BMSCs at passages 3–6 were used to generate conditioned media. Chrysin (Cat. No. HY-14589, MCE, USA) was prepared as a 10 mM stock solution in dimethyl sulfoxide (DMSO, Cat. No. HY-Y0320C, MCE, USA) and diluted in DMEM/F12 to the working concentration. The final DMSO concentration in culture was kept below 0.01%. BMSCs were pretreated with chrysin for 48 h. After pretreatment, cells were washed repeatedly with sterile PBS to remove residual chrysin and then incubated in fresh 30 mM glucose serum-free HG DMEM for 24 h to collect CM. Supernatants were collected, centrifuged at 300 *g* × 5 min, and filtered through a 0.22 μm sterile membrane to obtain Chrysin-CM. BMSC-conditioned medium (BMSC-CM) was collected in parallel using the same procedure without chrysin pretreatment. Conditioned media were stored at −80 °C until use.

To ensure experimental consistency, the conditioned media were generated under identical conditions, including the same initial cell seeding density, culture volume, conditioning duration, and collection procedures across groups. For in vitro experiments, the collected conditioned media were applied directly to the target cells using the same volume across experimental groups.

### 2.4. Cell Viability and Proliferation Assays

Cell viability was assessed using the CCK-8 assay (Cat. No. A311-01, Vazyme, Nanjing, China), following the manufacturer’s instructions. Briefly, cells were seeded into 96-well plates at an appropriate density and incubated with CCK-8 working solution for 2 h at 37 °C. The absorbance at 450 nm was measured using a microplate reader (BioTek Instruments, Winooski, VT, USA) to evaluate the cellular metabolic activity, which reflects the overall cell viability.

Cell proliferation was evaluated using a 5-ethynyl-2′-deoxyuridine (EdU) incorporation assay (Cat. No. A411-01, Vazyme, China). After 48 h of treatment with chrysin, BMSCs were incubated with EdU reagent for 2 h, fixed with 4% paraformaldehyde, and stained, according to the manufacturer’s protocol. The nuclei were specifically stained with 4′,6-diamidino-2-phenylindole (DAPI), and EdU-positive cells were visualized and quantified under a fluorescence microscope (Olympus, Tokyo, Japan) to determine the proliferation index.

### 2.5. Cell Migration Assays

Cell migration ability was assessed using both scratch wound-healing and transwell migration assays. For the scratch wound-healing assay, cells were seeded in six-well plates and cultured to approximately 90% confluence. A linear wound was created using a sterile 200 μL pipette tip, and detached cells were gently removed by washing twice with PBS. The cells were then incubated in serum-free medium under the indicated treatment conditions. Images of the wound area were captured at 0 h and 24 h using an inverted microscope (Olympus, Japan), and the wound closure percentage was quantified using the ImageJ software (version 1.54g, NIH, Bethesda, MD, USA).

For the transwell migration assay, 24-well transwell chambers with 8 μm pore polycarbonate membranes (Corning, Steuben County, NY, USA) were used. Cells were seeded into the upper chamber in serum-free medium, while the lower chamber contained medium supplemented with 10% FBS as a chemoattractant. After incubation for 24 h at 37 °C, non-migrated cells were removed from the upper surface with a cotton swab, and the migrated cells on the lower membrane surface were fixed with 4% paraformaldehyde for 15 min and stained with 0.5% crystal violet. Images were acquired under a light microscope (Olympus, Japan), and migrated cells were quantified by counting in randomly selected fields.

### 2.6. Tube Formation Assay

The angiogenic capacity of endothelial cells was evaluated using a tube formation assay. Briefly, 200 μL of growth factor-reduced Matrigel (BD Biosciences, USA) was added to each well of a 24-well plate and allowed to polymerize at 37 °C for 30 min. HUVECs were then seeded onto the solidified Matrigel (Cat. No. BL1834, Biosharp, Beijing, China) at a density of 2 × 10^4^ cells per well and cultured under the indicated treatment conditions for 6 h.

After incubation, capillary-like tube structures were visualized using an inverted phase-contrast microscope (Olympus, Japan). Tube formation was quantitatively analyzed using the ImageJ software (NIH, USA) with the Angiogenesis Analyzer plugin.

### 2.7. Collagen Synthesis Assay

Collagen synthesis was evaluated by quantifying the hydroxyproline (HYP) content using a commercial HYP assay kit (Cat. No. BC0250, Solarbio, Beijing, China), according to the manufacturer’s instructions. Briefly, cells were collected and lysed after treatment under the indicated conditions. The lysates were hydrolyzed with an acid solution at 95 °C for 20 min and neutralized before reaction with the chromogenic reagent. The HYP content was calculated based on the standard curve and normalized to the total protein concentration, reflecting the collagen synthesis capacity of fibroblasts.

### 2.8. Oxidative Stress Assay

The intracellular reactive oxygen species (ROS) levels were determined using the fluorescent probe 2′,7′-dichlorofluorescein diacetate (DCFH-DA; Cat. No. D6470, Solarbio, China). After treatment, cells were incubated with 10 μM DCFH-DA at 37 °C for 1 h in the dark, washed 3 times with serum-free medium, and immediately observed under a fluorescence microscope (Olympus, Japan). The mean fluorescence intensity was quantified using the ImageJ software (NIH, USA) to evaluate the intracellular ROS accumulation.

Lipid peroxidation was assessed by measuring malondialdehyde (MDA) levels using a lipid peroxidation (MDA) assay kit (Cat. No. ADS-F-YH002, Jiangsu Addison Biological Technology Co., Ltd., Suzhou, China), according to the manufacturer’s protocol. Cell lysates were mixed with thiobarbituric acid (TBA) reagent, boiled for 15 min, and centrifuged at 1000× *g* for 10 min. The absorbance of the supernatant was recorded at 532 nm, and the MDA levels were normalized to the total protein content.

### 2.9. Cell Cycle Analysis

The cell cycle distribution was analyzed via flow cytometry using propidium iodide (PI) staining. BMSCs were harvested after the indicated treatments, washed twice with PBS, and fixed in 70% cold ethanol at 4 °C overnight. After fixation, the cells were washed again with PBS and incubated with RNase A and propidium iodide (Cat. No. IP50308, Solarbio, China) for 30 min at room temperature in the dark.

The fluorescence intensity was measured using a flow cytometer (BD FACSCalibur, San Jose, CA, USA), and data were analyzed with the FlowJo software (version 10.8.1, Tree Star, Ashland, OR, USA). The percentages of cells in the G_0_/G_1_, S, and G_2_/M phases were calculated to evaluate the effects of chrysin on cell cycle progression in BMSCs.

### 2.10. Diabetic Wound Model and Treatment in Mice

To establish the diabetic wound model, male C57BL/6 mice aged 8 weeks were rendered diabetic using an intraperitoneal 50 mg/kg STZ (Cat. No. BS185, Biosharp, China) injection for 5 consecutive days. STZ was freshly dissolved in 0.1 M citrate buffer at pH 4.5 immediately before each injection. Hyperglycemia was confirmed when the fasting blood glucose levels exceeded 16.7 mmol/L at 7 days after the final injection. For the subsequent surgical procedure, mice were anesthetized with isoflurane using an inhalation anesthesia system, with 2% isoflurane for induction and 1.5–2% for maintenance throughout surgery. After hair removal and skin disinfection, a full-thickness excisional wound with a diameter of 10 mm was created on the dorsal skin using a biopsy punch. Mice were randomly assigned to 3 groups, with 6 mice in each group. The control group received 100 μL of sterile PBS (Cat. No. 10010023, Gibco, USA), the CM group received BMSC-CM, and the Chrysin-CM group received Chrysin-CM.

Each wound received 100 μL of the corresponding treatment via perilesional injection once daily. The total volume was delivered through five injections, with four evenly distributed around the wound margin and one in the center of the wound. The wound closure was monitored using digital photography on days 0, 3, 7, 10, and 14. A scale reference was included in each image, and the wound margins were manually traced using ImageJ (NIH, USA) for quantitative analysis.

All animal experiments were performed at the Animal Experimental Center of the First Clinical College, Wuhan University. All surgical procedures were performed under isoflurane anesthesia. Mice were housed in a temperature- and humidity-controlled environment under a 12 h light/dark cycle, with free access to standard chow and water. Histological and morphometric assessments were performed by investigators blinded to the group allocation. No animals or data points were excluded from the analysis during the study.

### 2.11. Histological Analysis

Wound tissues were harvested on days 7 and 14, fixed in 4% paraformaldehyde, and embedded in paraffin. Sections were stained with hematoxylin and eosin (H&E) for re-epithelialization and with Masson’s trichrome for collagen deposition. Immunohistochemical (IHC) staining for CD31 (Cat. No. 11265-1-AP, Proteintech, Rosemont, IL, USA) was performed to assess neovascularization, and positive areas were quantified using ImageJ. IHC staining for Ki-67 (Cat. No. 27309-1-AP, Proteintech, USA) was performed to assess the proliferative activity in wound tissues, and the percentage of Ki-67-positive cells was quantified using ImageJ.

### 2.12. Immunofluorescence (IF) Staining

Wound tissues were harvested, fixed in 4% paraformaldehyde, embedded in paraffin, and sectioned. After deparaffinization and antigen retrieval, sections were blocked with 5% bovine serum albumin (BSA) and incubated overnight at 4 °C with a primary antibody against Ly6G (Cat. No. 65140-1-Ig, Proteintech, USA). After washing with PBS, sections were incubated with the corresponding fluorescent secondary antibody at room temperature for 1 h in the dark. Nuclei were counterstained with DAPI. Fluorescence images were captured using a fluorescence microscope, and the fluorescence intensity of Ly6G-positive signals was quantified using ImageJ.

### 2.13. RNA-Seq and Bioinformatic Analysis

Total RNA from wound tissues was extracted using TRIzol reagent (Cat. No. R1100, Solarbio, China), RNA integrity was confirmed using an Agilent 2100 Bioanalyzer, and sequencing was performed on an Illumina NovaSeq 6000 platform. Differentially expressed genes (DEGs) were identified with the DESeq2 software (version 1.50.2) using |log_2_FC| > 1 and FDR < 0.05 as thresholds. Gene Ontology (GO), Kyoto Encyclopedia of Genes and Genomes (KEGG), and gene set enrichment analysis (GSEA) were performed using ClusterProfiler. Immune cell infiltration was analyzed using Cell-type Identification by Estimating Relative Subsets of RNA Transcripts (CIBERSORT), and alternative splicing (AS) events were identified with the replicate Multivariate Analysis of Transcript Splicing (rMATS) software (version 4.3.0).

### 2.14. Western Blot

Wound tissues were homogenized in RIPA lysis buffer containing protease and phosphatase inhibitors to extract the total protein. Protein concentrations were determined using a BCA protein assay kit. Equal amounts of protein were separated via SDS-PAGE and transferred onto PVDF membranes. After blocking with 5% non-fat milk for 1 h at room temperature, membranes were incubated overnight at 4 °C with primary antibodies against p38 (Cat. No. R25239, ZenBio, Chengdu, China), NF-κB p65 (Cat. No. R380172, ZenBio, Chengdu, China), and β-tubulin (Cat. No. 14555-1-AP, Proteintech, USA). After washing with TBST, membranes were incubated with HRP-conjugated secondary antibodies at room temperature for 1 h. Protein bands were visualized using an enhanced chemiluminescence (ECL) detection system and quantified using ImageJ. β-tubulin was used as the loading control.

### 2.15. Statistical Analysis

All quantitative data are presented as the mean ± standard deviation (M ± SD), based on at least three independent experiments. Statistical analyses were performed using GraphPad Prism (version 10.2.1, GraphPad Software, Boston, MA, USA). Data distribution and variance assumptions were evaluated before parametric testing. For comparisons between two groups, the unpaired Student’s *t*-test was applied. For comparisons among three or more groups, one-way analysis of variance (ANOVA) followed by Bonferroni’s multiple-comparison post hoc test was used. In the wound-healing assay, two-way ANOVA was performed to evaluate the effects of treatment and time. Statistical significance was identified as *p* < 0.05, indicated by asterisks as follows: * *p* <  0.05, ** *p* <  0.01, *** *p* <  0.005, and **** *p* <  0.001.

## 3. Results

### 3.1. Isolation and Identification of Primary BMSCs

**Figure 1:**
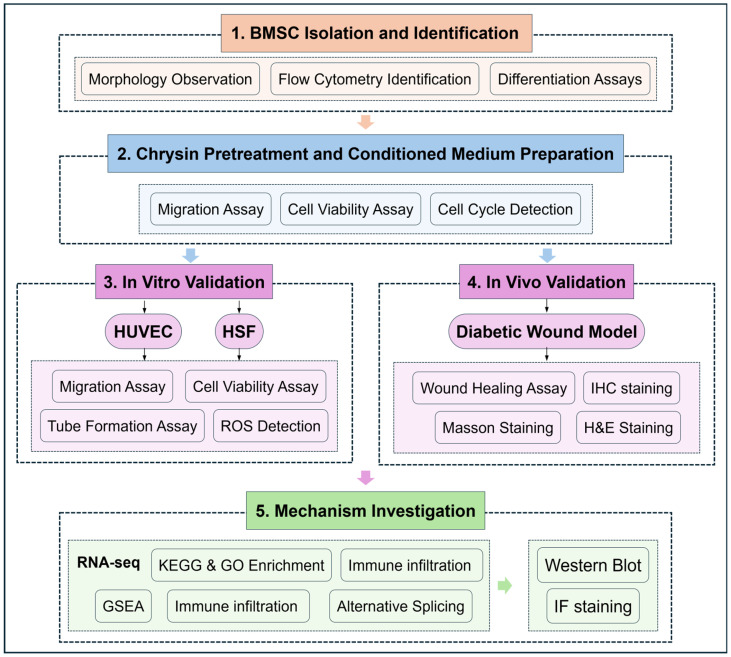
Schematic illustration of the study workflow.

**Figure 2:**
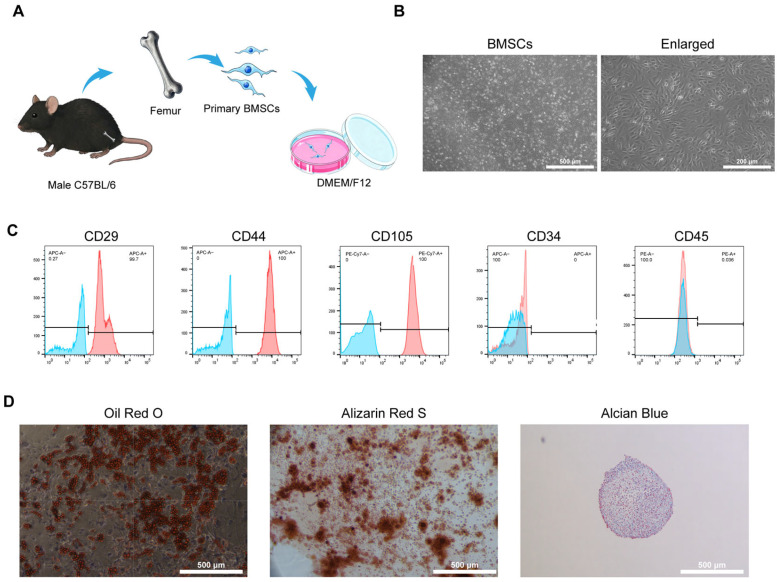
Isolation and identification of primary BMSCs from C57BL/6 mice. (**A**) Schematic diagram showing the isolation process of BMSCs from C57BL/6 mouse femurs; (**B**) Morphological observation of BMSCs under a light microscope; (**C**) Flow cytometric analysis of BMSC surface markers. Representative histograms showing expression of mesenchymal markers (CD29, CD44, and CD105) and hematopoietic markers (CD34 and CD45). The blue peak indicates the isotype (negative) control, and the red peak indicates the antibody-stained BMSC population used for marker identification. (**D**) Trilineage differentiation of BMSCs. Oil-red O staining of adipogenesis differentiation of BMSCs. Alizarin red staining of osteogenic differentiation of BMSCs. Alcian blue staining of chondrogenic differentiation of BMSCs.

Figure 1 provides an overview of the experimental workflow of the study. Primary BMSCs were isolated from the femurs of C57BL/6 mice (Figure 2A). The extracted cells exhibited a typical spindle-shaped and fibroblast-like morphology under the optical microscope after adherent culture (Figure 2B). Flow cytometric analysis demonstrated high expression levels of the mesenchymal surface markers CD29, CD44, and CD105. Conversely, the expression of hematopoietic lineage markers, including CD34 and CD45, was negligible, confirming the characteristic mesenchymal stem cell phenotype (Figure 2C). The multipotent differentiation potential of BMSCs was further verified via lineage-specific staining (Figure 2D). Following adipogenic induction, Oil Red O staining identified distinct red-stained lipid droplets within the cytoplasm of the cells. Alizarin Red S staining indicated the presence of extensive red-orange calcium deposits in osteogenic cultures. Finally, Alcian Blue staining confirmed cartilage matrix formation during chondrogenic differentiation, which was visible as dense blue-stained sulfated proteoglycans within the cell pellets. Therefore, we successfully isolated BMSCs and confirmed their phenotypes.

### 3.2. Effects of Chrysin on BMSC Proliferation, Migration, and Cell Cycle

**Figure 3:**
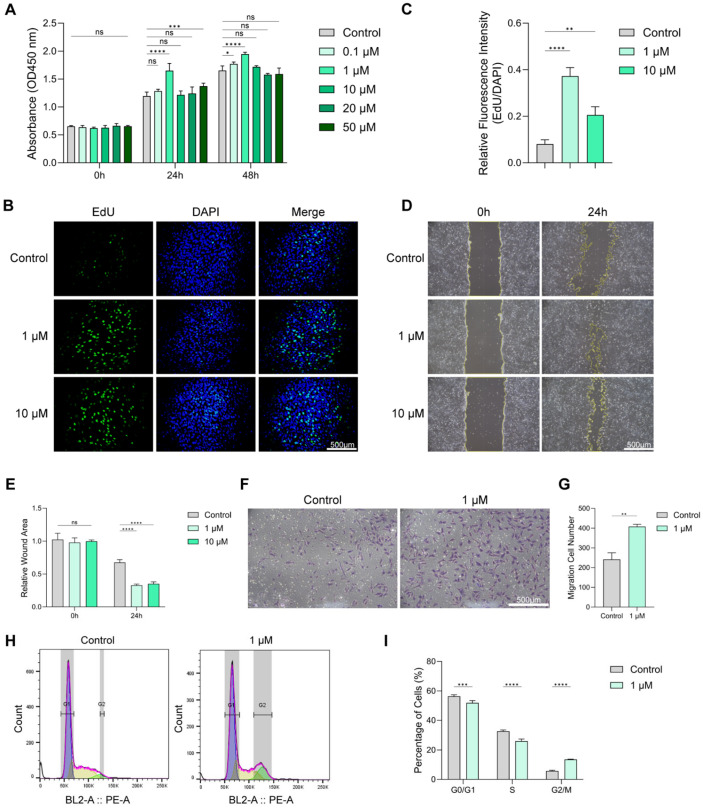
Effects of chrysin on the proliferation, migration, and cell cycle of BMSCs. (**A**) CCK-8 assay of BMSCs treated with different concentrations of Chrysin at 0.1, 1, 10, 20, or 50 μM for 0, 24, and 48 h. (**B**,**C**) EdU staining and quantitative analysis of BMSCs after treatment with 1 μM and 10 μM Chrysin. Scale bar = 500 μm. (**D**,**E**) Scratch wound-healing assay and quantification of BMSC migration after treatment with 1 μM and 10 μM Chrysin for 24 h. Scale bar = 500 μm. (**F**,**G**) Transwell migration assay and quantification of migrated BMSCs treated with 1 μM Chrysin. Scale bar = 250 μm. (**H**,**I**) Flow cytometric analysis of cell cycle distribution and corresponding statistical results of BMSCs after treatment with 1 μM Chrysin (ns, not significant; * *p* < 0.05, ** *p* < 0.01, *** *p* < 0.001, **** *p* < 0.0001).

We examined the effect of the chrysin concentration on the viability of BMSCs using the CCK-8 assay. The treatment with 1 μM chrysin significantly increased cellular viability after 24 h and 48 h compared with the Control group, whereas higher concentrations of chrysin slightly inhibited the cellular viability (Figure 3A). Compared to the Control group, BMSCs treated with 1 μM or 10 μM chrysin demonstrated significantly higher EdU fluorescence intensity (Figure 3B,C). This observation suggested that chrysin effectively enhanced cell proliferation. Similarly, scratch wound-healing assays revealed that 1 μM and 10 μM chrysin treatment accelerated wound closure after 24 h, suggesting the improved migratory ability of BMSCs (Figure 3D,E). Based on these results, 1 μM chrysin for 48 h was chosen as the optimal pretreatment condition for subsequent experiments.

Transwell assays further verified the pro-migratory effect of chrysin, showing a higher number of migrated BMSCs in the 1 μM group compared with the Control group (Figure 3F,G). Flow cytometric analysis showed that chrysin treatment increased the proportion of BMSCs in the G2/M phase while decreasing the fractions in the G0/G1 and S phases, indicating altered cell-cycle distribution (Figure 3H,I). Collectively, these results suggest that low-dose chrysin enhances BMSC proliferation and migration and is associated with a shift in cell cycle distribution toward the G2/M phase.

### 3.3. Chrysin-CM Protects HUVECs from HG-Induced Injury

Considering that the biological functions of BMSCs are closely related to their secreted factors, HUVECs were divided into 4 groups to assess the effects of Chrysin-CM on endothelial function. HUVECs in the Control group were cultured in normal-glucose medium, while the other three groups were exposed to HG medium alone, HG medium supplemented with BMSC-CM, or HG medium supplemented with Chrysin-CM.

**Figure 4:**
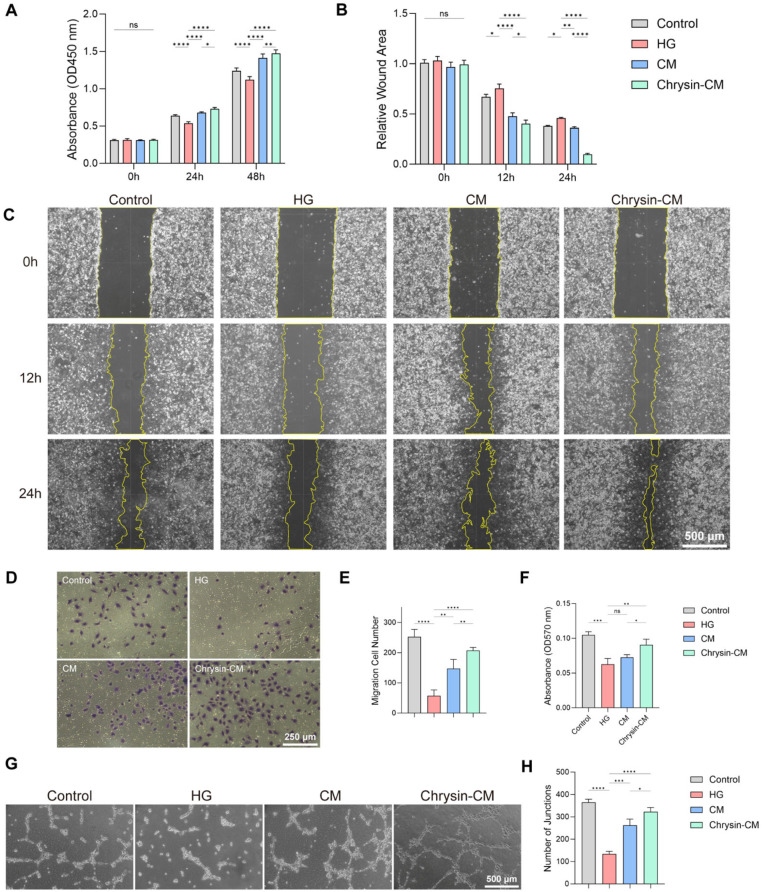
Chrysin-CM protects HUVECs from HG-induced injury. HUVECs were treated with control, HG, BMSC-CM, or Chrysin-CM. (**A**) CCK-8 assay of HUVEC viability. (**B**,**C**) Scratch wound-healing assay and quantification of HUVEC migration. Scale bar = 500 μm. (**D**,**E**) Transwell migration assay and quantification of migrated HUVECs. Scale bar = 250 μm. (**F**–**H**) Tube formation assay and quantification of angiogenic structures. Scale bar = 500 μm (ns, not significant; * *p* < 0.05, ** *p* < 0.01, *** *p* < 0.001, **** *p* < 0.0001).

The CCK-8 assay showed that HG exposure reduced HUVEC proliferation, while both BMSC-CM and Chrysin-CM significantly improved viability, with the latter showing the most pronounced effect (Figure 4A). Scratch wound-healing assays demonstrated that the HG condition impaired endothelial migration, while BMSC-CM partially restored and Chrysin-CM further enhanced the migratory ability of HUVECs (Figure 4B,C). Consistently, transwell migration assays confirmed a higher number of migrated cells following treatment with BMSC-CM and Chrysin-CM (Figure 4D–F). Furthermore, tube formation assays revealed that Chrysin-CM markedly promoted the angiogenic capacity of HUVECs, as indicated by increased tube-like structures and higher junction counts, when compared with the other groups (Figure 4G,H). These findings indicate that Chrysin-CM effectively restored the proliferation, migration, and angiogenic potential of HUVECs impaired by HG induction.

### 3.4. Chrysin-CM Protects HSFs from HG-Induced Injury

Following the experiments on endothelial cells, we next investigated the effects of Chrysin-CM on fibroblasts, another key cell type involved in wound healing. HSFs were divided into four experimental groups: control cultured in normal-glucose medium, and three HG groups treated with HG medium alone, BMSC-CM, and Chrysin-CM.

**Figure 5:**
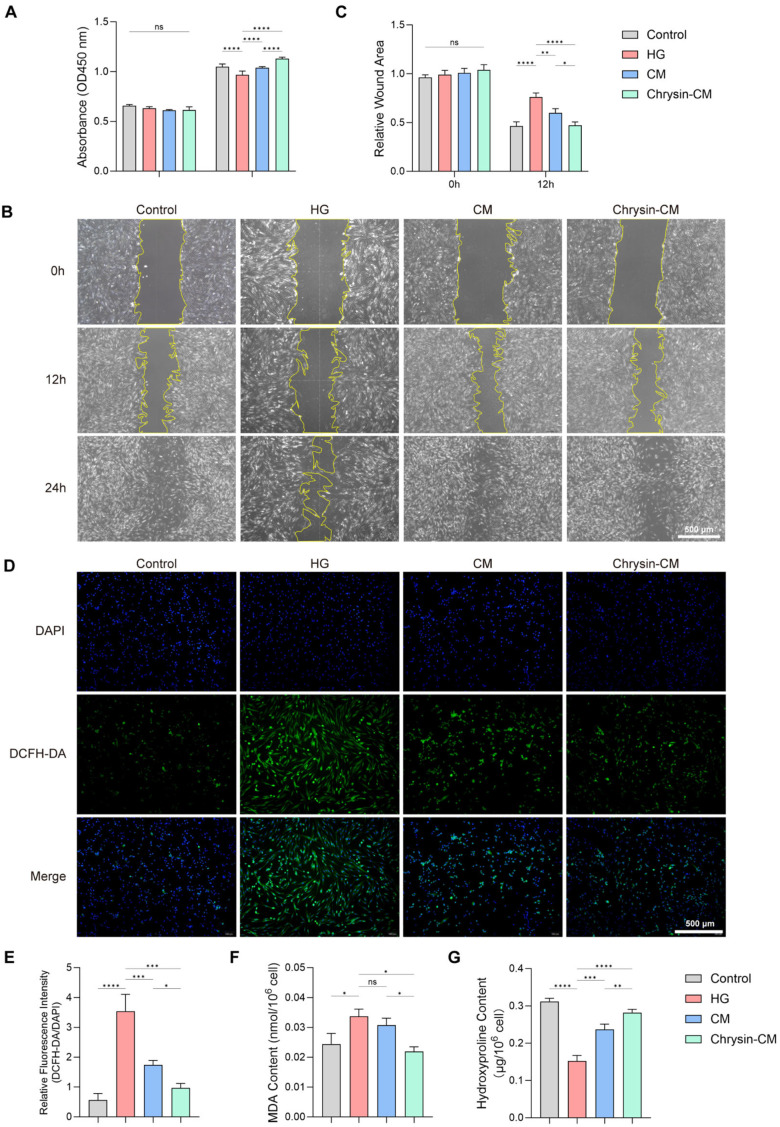
Chrysin-CM protects HSFs from HG-induced injury. HSFs were treated with control, HG, BMSC-CM, or Chrysin-CM. (**A**) CCK-8 assay of HSF viability. (**B**,**C**) Scratch wound-healing assay and quantification of fibroblast migration. Scale bar = 500 μm. (**D**,**E**) DCFH-DA staining and quantification of ROS fluorescence. (**F**) Measurement of intracellular MDA content. (**G**) Determination of HYP content (ns, not significant; * *p* < 0.05, ** *p* < 0.01, *** *p* < 0.001, **** *p* < 0.0001).

The CCK-8 assay showed that HG induction significantly reduced HSF proliferation, whereas both BMSC-CM and Chrysin-CM markedly improved cell viability, with the latter showing the strongest effect (Figure 5A). The wound-healing assay revealed that the fibroblast migration was reduced under HG conditions. Treatment with BMSC-CM promoted wound closure, and Chrysin-CM further accelerated this process (Figure 5B,C). To evaluate the oxidative stress levels, we then performed DCFH-DA staining in HSFs and found that the ROS levels were elevated under HG conditions. This increase was reduced in cells treated with BMSC-CM and further alleviated in those treated with Chrysin-CM (Figure 5D,E). Consistently, Chrysin-CM significantly decreased the intracellular MDA content (Figure 5F). Moreover, HYP quantification showed an increase in collagen synthesis following treatment with Chrysin-CM (Figure 5G).

These findings verified that Chrysin-CM effectively protected HSFs from HG-induced oxidative damage with enhanced proliferation, migration, and matrix-forming capacity.

### 3.5. Chrysin-CM Accelerates Wound Healing in Diabetic Mice

**Figure 6:**
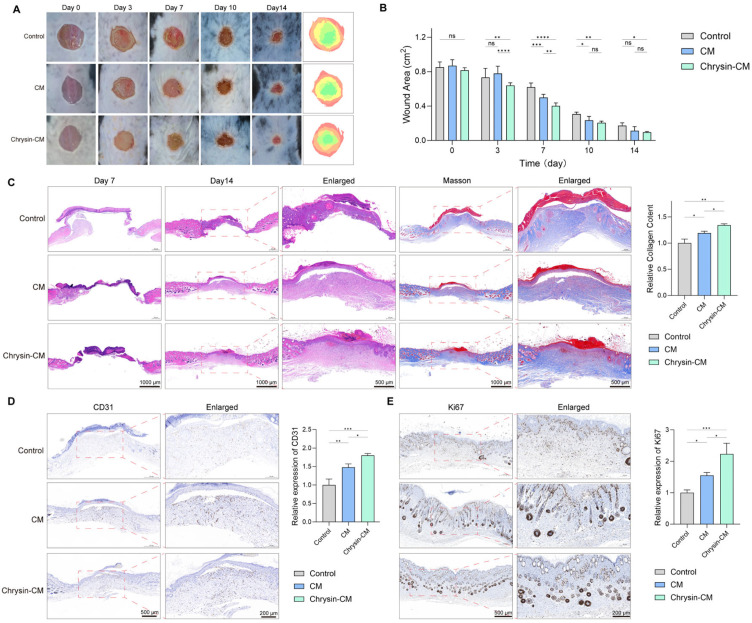
Effects of Chrysin-CM on wound healing in diabetic mice. Diabetic mice were treated with control, BMSC-CM, or Chrysin-CM. (**A**) Representative images of wound healing at days 0, 3, 7, 10, and 14. (**B**) Quantitative analysis of relative wound area at each time point with between-group statistical comparisons indicated. (**C**) H&E staining of wound sections at day 7 and day 14. Masson’s trichrome staining with quantitative analysis. Scale bar = 1000 μm and 500 μm. (**D**) CD31 immunohistochemistry of wound sections with quantitative analysis. Scale bar = 500 μm and 200 μm. (**E**) Representative Ki-67 immunohistochemistry images of wound sections with quantitative analysis. Scale bar = 500 μm and 200 μm (ns, not significant; * *p* < 0.05, ** *p* < 0.01, *** *p* < 0.001, **** *p* < 0.0001.).

Wound healing was evaluated in diabetic mice treated with control medium, BMSC-CM, or Chrysin-CM. Both BMSC-CM and Chrysin-CM accelerated wound closure compared with the control group, as demonstrated by macroscopic observation and quantitative analysis of the wound area (Figure 6A,B). Notably, Chrysin-CM exhibited a more pronounced effect during the early phase of healing, showing significantly smaller wound areas than BMSC-CM during the initial stage of the wound-healing process. Histological assessment allowed for further assessment of the efficacy of wound healing in the Chrysin-CM group. H&E staining demonstrated more complete re-epithelialization and better overall tissue organization at both day 7 and day 14, and Masson’s trichrome staining showed increased collagen deposition through quantitative analysis (Figure 6C). Consistently, CD31 IHC with quantitative analysis revealed enhanced neovascularization in the Chrysin-CM group (Figure 6D).

In addition, the IHC results showed that there was increased Ki-67 expression in the Chrysin-CM group (Figure 6E). Collectively, these findings indicated that Chrysin-CM promoted diabetic wound healing by enhancing re-epithelialization, collagen regeneration, angiogenesis, and cellular proliferation.

### 3.6. Transcriptomic Analysis of Wound Tissues Treated with Chrysin-CM

**Figure 7:**
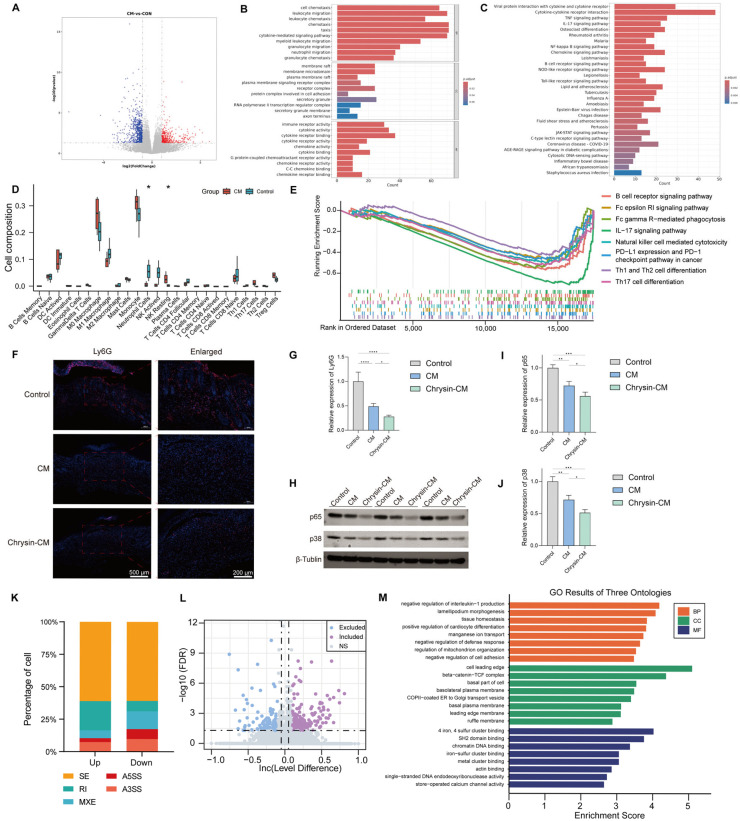
Transcriptomic analysis of wound tissues after Chrysin-CM treatment. RNA sequencing was performed on wound tissues collected from control and Chrysin-CM–treated diabetic mice on day 14. (**A**) Volcano plot of DEGs, red indicates significantly upregulated genes, blue indicates significantly downregulated genes, and gray indicates genes without significant differential expression; (**B**,**C**) GO and KEGG enrichment analyses of DEGs; (**D**) Immune cell infiltration profiles; (**E**) GSEA of immune-related pathways; (**F**) Representative immunofluorescence staining of Ly6G; (**G**) Quantification of Ly6G-positive signal intensity; (**H**) Representative Western blot images showing p65 and p38 protein levels in wound tissues; (**I**,**J**) Densitometric quantification of p65 and p38 normalized to β-Tubulin; (**K**) Proportional distribution of differential AS event types among upregulated and downregulated events, including skipped exon (SE), retained intron (RI), mutually exclusive exons (MXE), alternative 5′ splice site (A5SS), and alternative 3′ splice site (A3SS). (**L**) Volcano plot of differential AS events (**M**) GO enrichment results of alternatively spliced genes (* *p* < 0.05, ** *p* < 0.01, *** *p* < 0.001, **** *p* < 0.0001).

To elucidate the molecular mechanisms underlying the observed in vivo wound-healing effects, RNA-seq was performed on wound tissues collected at day 14 after treatment. Differential expression analysis identified 1022 significantly altered genes (|log_2_FC| > 1, FDR < 0.05), including 400 upregulated and 622 downregulated genes (Figure 7A). Functional enrichment analysis revealed that the differentially expressed genes were primarily enriched in processes related to cell migration, cytokine signaling, and immune regulation. GO terms such as chemotaxis, leukocyte migration, and cytokine-mediated signaling, along with KEGG pathways including IL-17 signaling, neutrophil migration, and NF-κB signaling, indicated that Chrysin-CM treatment inhibited inflammatory activation and optimized immune responses for tissue repair (Figure 7B,C).

Immune infiltration analysis further supported these findings, showing reduced neutrophil and activated natural killer (NK) cell infiltration in the Chrysin-CM group compared with the Control group (Figure 7D). The GSEA result demonstrated overall downregulation of inflammatory and adaptive immune pathways, including IL-17 signaling, Th17 differentiation, and NK cell-mediated cytotoxicity, consistent with the anti-inflammatory effect of Chrysin-CM (Figure 7E). Consistent with these findings, RNA-seq analysis also identified the downregulation of Cd177, a neutrophil-associated marker, in the Chrysin-CM group. To experimentally validate the transcriptomics-predicted reduction in neutrophil infiltration, IF staining for Ly6G was performed on wound sections. Ly6G-positive signals were markedly decreased in the Chrysin-CM group, and the quantitative analysis confirmed reduced Ly6G expression (Figure 7F,G). In addition, Western blotting of wound tissues demonstrated decreased protein levels of NF-κB p65 and p38 MAPK in the Chrysin-CM group (Figure 7H–J). AS analysis revealed that Chrysin-CM modulated RNA splicing events in genes associated with wound healing, including processes such as negative regulation of interleukin-1 production, lamellipodium morphogenesis, and actin filament organization. KEGG enrichment of alternatively spliced genes further implicated pathways involved in cell adhesion and immune regulation, such as adherens junctions and Fc gamma receptor (FCγR)-mediated phagocytosis (Figure 7K–M).

Overall, the RNA-seq analysis indicated that Chrysin-CM was associated with the attenuation of inflammatory and immune-related pathways in diabetic wounds, further supported by the reduction in Ly6G-positive neutrophils and the decreased protein expression of p65 and p38.

## 4. Discussion

BMSC-based therapies from multiple sources have become a hot research topic for the treatment of diabetic wounds [11]. Accordingly, considerable effort has been devoted to improving the therapeutic efficacy of BMSCs. For example, MG53-overexpressing BMSCs enhanced diabetic wound healing through activation of the eNOS/NO pathway [12]. Similarly, Zheng et al. constructed Insig1-overexpressing BMSCs which enhanced the secretion of miR-132-3p-enriched exosomes, thereby promoting wound healing in diabetic mice [13]. However, these enhancement strategies have largely depended on genetic engineering approaches, which may increase the experimental complexity and translational barriers.

In this context, pretreating BMSCs with physical, chemical, or biological stimuli represents a simpler and potentially more practical strategy to improve the biological activity and regenerative efficacy of BMSCs without the need for recombinant cytokine supplementation or permanent genetic manipulation [14]. Yi et al. demonstrated that AA2G-pretreated BMSCs exhibited enhanced proliferation and angiogenic capacity, and their CM promoted fibroblast migration and collagen synthesis. Mechanistically, AA2G activated the PI3K/AKT pathway, upregulated VEGF, and enhanced TET2-mediated DNA demethylation, thereby accelerating wound healing and neovascularization in vivo [15]. In addition, δ-Tocotrienol pretreatment improved the regenerative potential of BMSCs by enhancing their pro-proliferative and pro-angiogenic effects and mitigating ferroptosis in target cells. This effect was associated with the suppression of BACH1 expression and the activation of the PI3K/AKT signaling pathway, ultimately accelerating wound healing in vivo [16]. Collectively, these studies show that the pharmacological preconditioning of BMSCs can significantly enhance their paracrine therapeutic potential before clinical application.

Among the various pharmacological pretreatment methods, small molecules derived from natural sources are highly favored due to their relative safety, ease of access, and multifaceted biological activity. Flavonoids are a class of widely occurring phytochemicals that are potent modulators of key signaling pathways essential for cell survival, proliferation, and paracrine secretion. Flavonoids promote MSC proliferation and osteogenic differentiation via activation of Wnt/β-catenin, PI3K/Akt, and MAPK/ERK signaling pathways, thereby improving regenerative outcomes [17]. Peng et al. reported that quercetin pretreatment enhanced the production and antioxidant cargo of MSC-derived exosomes, thereby improving their anti-inflammatory and regenerative effects through inhibition of NLRP3 inflammasome activation and pyroptosis in nucleus pulposus cells [18]. Similarly, apigenin has been reported to enhance the antioxidant capacity and osteogenic differentiation of osteoporotic BMSCs by restoring redox homeostasis through the PI3K/Akt pathway [19]. These studies showed that flavonoids could enhance the reparative potential of MSC-based or regenerative strategies through modulation of inflammation, oxidative stress, and tissue remodeling. Based on these observations, we speculated that chrysin, another bioactive flavonoid, might be able to enhance the biological activity and therapeutic potential of BMSCs. In the present study, chrysin pretreatment enhanced the biological activity of BMSCs, and Chrysin-CM promoted endothelial and fibroblast functions relevant to wound healing. Moreover, Chrysin-CM accelerated diabetic wound healing, possibly by attenuating neutrophil-associated inflammation and suppressing IL-17-related inflammatory signaling.

The RNA sequencing analysis of the wound tissues provided molecular evidence that Chrysin-CM reshaped the diabetic wound microenvironment. Transcriptomic analysis identified broad gene expression changes, characterized by 1022 differentially expressed genes, with GO and KEGG enrichment analyses suggesting the downregulation of inflammation-related pathways, including IL-17 signaling, neutrophil migration, and NF-κB signaling. In parallel, immune infiltration analysis indicated reduced neutrophil- and activated NK cell-associated signatures in the Chrysin-CM group compared with the Control group. Furthermore, GSEA supported a coordinated reduction in immune and inflammatory pathway activities. Together, these results highlighted neutrophil-associated inflammation and IL-17-related immune activation as potential processes influenced by Chrysin-CM. Therefore, based on these transcriptomic findings, we performed protein-level validation focusing on neutrophil infiltration and inflammatory signaling. Consistent with the predicted inflammatory attenuation, Ly6G IF showed decreased neutrophil accumulation in the Chrysin-CM group. Furthermore, Western blotting demonstrated reduced protein levels of NF-κB p65 and p38 MAPK. Collectively, these findings provided protein-level support that Chrysin-CM was associated with reduced neutrophil-linked inflammation and the attenuation of related inflammatory signaling in vivo.

Alongside these pathway-level shifts, several DEGs may be relevant to the observed phenotype and warrant further investigation. Among the most prominently downregulated transcripts were Fos and Fosb, immediate early response genes that have been implicated in sustained inflammation and delayed healing in diabetes [20]. Cd177 was also identified as a downregulated transcript in the RNA-seq dataset. Given its reported association with neutrophil recruitment and activation, this finding is consistent with the decreased neutrophil-related signatures suggested by immune infiltration analysis and our Ly6G-based validation [21]. In addition, the most significantly upregulated genes included Adrb3 and Tshr. The upregulation of Adrb3 may be associated with enhanced local vasodilation and angiogenesis, which could improve blood and nutrient supply during tissue repair [22]. Cianfarani et al. reported that TSHR upregulation suggests promotion of keratinocyte proliferation and differentiation, potentially contributing to re-epithelialization [23]. While these associations remain inferential at present, they offer plausible and testable hypotheses linking transcriptomic shifts to coordinated programs of vascularization and epithelial repair during wound healing. Additionally, the AS analysis suggested a post-transcriptional layer of regulation that may be relevant to wound healing. AS events were enriched in processes such as lamellipodium morphogenesis, tissue homeostasis, and actin filament organization, and the pathway analysis of AS genes highlighted associations with adherens junctions and FCγR-mediated phagocytosis pathways. These findings may indicate that Chrysin-CM influences transcript isoform usage in pathways related to cytoskeletal dynamics, cell–cell interactions, and immune-related functions.

However, several limitations of the present study should be acknowledged. Firstly, the in vitro experiments were performed under HG (30 mM) conditions to reproduce hyperglycemia-associated cellular dysfunction relevant to the diabetic wound pathology. Although this model is widely used to simulate sustained hyperglycemic stress, it represents a simplified system and does not fully capture the complex in vivo microenvironment of diabetic wounds, which involves coordinated interactions among inflammatory responses, oxidative stress, hypoxia, vascular impairment, and ECM remodeling. Secondly, although our results demonstrated that chrysin enhanced the therapeutic efficacy of BMSC-CM, we did not systematically characterize the compositional changes in the secretome induced by chrysin pretreatment. CM represents a complex mixture of bioactive component which may include soluble proteins such as cytokines and growth factors, lipids and small signaling molecules, nucleic acids, and extracellular vesicles. Therefore, the observed therapeutic effects are likely mediated by the coordinated actions of multiple factors, rather than a single molecule. A comprehensive analysis would require multi-layered approaches, such as quantitative proteomics to identify altered soluble factors, lipidomic or metabolomic profiling to detect small molecules, and extracellular vesicle isolation combined with RNA or protein characterization. Such integrated multi-omics strategies, followed by the functional validation of candidate mediators, will be important directions for future research. Finally, although the transcriptomic analysis provided insight into pathways potentially involved in immune regulation and tissue repair, some bioinformatic findings require further experimental confirmation. For example, the alternative splicing events identified in this study were not functionally investigated, and their precise roles in diabetic wound healing remain to be determined in future studies.

## 5. Conclusions

Our study demonstrated that pharmacological pretreatment with chrysin improved the therapeutic efficacy of BMSC-CM. Chrysin-CM effectively accelerated diabetic wound healing by inhibiting inflammation, reducing oxidative stress, and promoting angiogenesis and ECM. This strategy provided a practical and scalable approach to improve stem cell-based regenerative therapy and highlighted the therapeutic potential of chrysin as a pharmacological enhancer for the acceleration of chronic diabetic wound healing.

## Data Availability

The datasets used and/or analyzed during the current study are available from the corresponding author upon request.

## Abbreviations

Ascorbic acid 2-glucoside (AA2G); Beta-3 adrenergic receptor (Adrb3); Analysis of variance (ANOVA); Alternative splicing (AS); BTB and CNC homology 1 (BACH1); Bone marrow-derived mesenchymal stem cells (BMSCs); Bone marrow mesenchymal stem cell-conditioned medium (BMSC-CM); Bovine serum albumin (BSA); Cluster of differentiation 177 (Cd177); Cell Counting Kit-8 (CCK-8); Cell-type Identification by Estimating Relative Subsets of RNA Transcripts (CIBERSORT); Chrysin-pretreated BMSC-conditioned medium (Chrysin-CM); Conditioned medium (CM); Carbon dioxide (CO_2_); 4′,6-diamidino-2-phenylindole (DAPI); 2′,7′-Dichlorofluorescein diacetate (DCFH-DA); Differentially expressed genes (DEGs); Diabetic foot ulcer (DFU); Dulbecco’s Modified Eagle Medium (DMEM); Dulbecco’s Modified Eagle Medium/Nutrient Mixture F-12 (DMEM/F12); Dimethyl sulfoxide (DMSO); Extracellular matrix (ECM); 5-Ethynyl-2′-deoxyuridine (EdU); Enzyme-linked immunosorbent assay (ELISA); Extracellular vesicle (EV); Fluorescence-activated cell sorting (FACS); Fold change (FC); Fetal bovine serum (FBS); Fc gamma receptor (FCγR); False discovery rate (FDR); Fluorescein isothiocyanate (FITC); Gene Expression Omnibus (GEO); Gene Ontology (GO); Gene Set Enrichment Analysis (GSEA); High-glucose (HG); Hypoxia-inducible factor 1-alpha (HIF-1α); Hematoxylin and eosin (H&E); High-performance liquid chromatography (HPLC); Human skin fibroblasts (HSFs); Human umbilical vein endothelial cell (HUVEC); Hydroxyproline (HYP); Immunofluorescence (IF); Immunohistochemistry (IHC); Interleukin (IL); Kyoto Encyclopedia of Genes and Genomes (KEGG); Phospholysine phosphohistidine inorganic pyrophosphate phosphatase (LHPP); Mitogen-activated protein kinase (MAPK); Malondialdehyde (MDA); Mesenchymal stem cells (MSCs); Nuclear factor kappa-light-chain-enhancer of activated B cells (NF-κB); Natural killer cell (NK cell); Nuclear factor erythroid 2-related factor 2 (Nrf2); Phosphate-buffered saline (PBS); Propidium iodide (PI); Phosphoinositide 3-kinase (PI3K); Quantitative real-time polymerase chain reaction (qRT-PCR); replicate Multivariate Analysis of Transcript Splicing (rMATS); RNA sequencing (RNA-seq); Reactive oxygen species (ROS); Runt-related transcription factor 2 (Runx2); Standard deviation (SD); Short-chain dehydrogenase/reductase family 16C member 5 (SDR16C5); Superoxide dismutase (SOD); Streptozotocin (STZ); Thiobarbituric acid (TBA); Ten-eleven translocation methylcytosine dioxygenase 2 (TET2); T helper 17 cell (Th17); Tumor necrosis factor (TNF); Thyroid-stimulating hormone receptor (Tshr); Vascular endothelial growth factor (VEGF); Western blot (WB).
